# Revealing Chemical Trends: Insights from Data-Driven Visualization and Patent Analysis in Exposomics Research

**DOI:** 10.1021/acs.estlett.4c00560

**Published:** 2024-08-30

**Authors:** Dagny Aurich, Emma L. Schymanski, Flavio de Jesus Matias, Paul A. Thiessen, Jun Pang

**Affiliations:** † Luxembourg Centre for Systems Biomedicine (LCSB), University of Luxembourg, 6 Avenue du Swing, Belvaux L-4367, Luxembourg; ‡ Faculty of Science, Technology and Medicine (FSTM), University of Luxembourg, 6 Avenue de la Fonte, L-4364 Esch-sur-Alzette, Luxembourg; § National Center for Biotechnology Information, National Library of Medicine, National Institutes of Health, Bethesda, Maryland 20894, United States

**Keywords:** chemical stripes, PubChem, cheminformatics, data visualization, exposomics, early warning system, patent analysis

## Abstract

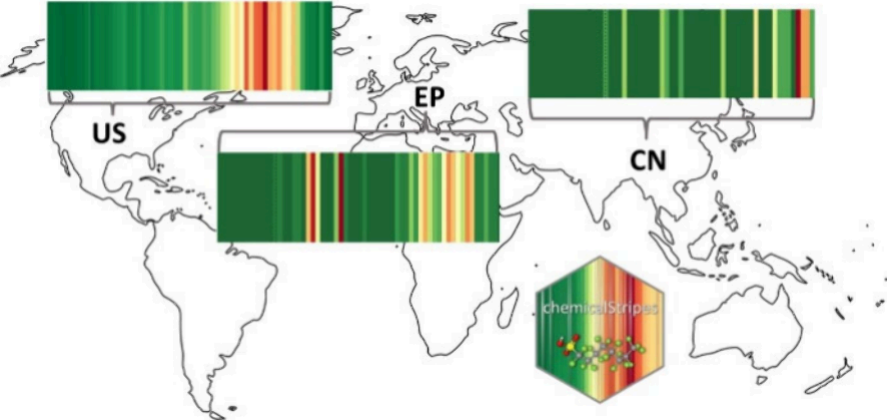

Understanding historical chemical usage is crucial for assessing current and past impacts on human health and the environment and for informing future regulatory decisions. However, past monitoring data are often limited in scope and number of chemicals, while suitable sample types are not always available for remeasurement. Data-driven cheminformatics methods for patent and literature data offer several opportunities to fill this gap. The *chemical stripes* were developed as an interactive, open source tool for visualizing patent and literature trends over time, inspired by the global warming and biodiversity stripes. This paper details the underlying code and data sets behind the visualization, with a major focus on the patent data sourced from PubChem, including patent origins, uses, and countries. Overall trends and specific examples are investigated in greater detail to explore both the promise and caveats that such data offer in assessing the trends and patterns of chemical patents over time and across different geographic regions. Despite a number of potential artifacts associated with patent data extraction, the integration of cheminformatics, statistical analysis, and data visualization tools can help generate valuable insights that can both illuminate the chemical past and potentially serve toward an early warning system for the future.

## Introduction

While studying historical and current chemical exposures can provide insights into their health and environmental impacts, the recreation of historical exposures to investigate past, present, or future health effects using analytical data is severely limited by several factors. These include the past focus on only a few dozen target chemicals (primarily legacy pollutants), in many cases a lack of suitable historical samples for remeasurement with modern analytical methods, and the sheer immensity of chemical space under consideration. Patent data, accessible through platforms like the World Intellectual Property Organization (WIPO) and linked to chemical structures in open databases such as PubChem,^1^ offers an alternative data-mining approach for examining past and potential chemical exposures, even though a mention in a patent does not always equate the use of a chemical.

PubChem is an open database of chemical structures, properties and associated information, providing tools for searching and analyzing chemical information.^1^ Approximately 40 million of the 118 million compounds in PubChem (June 2024) are linked to ∼51 million patent files.^2^ This Google Patents data set covers 120 million patent publications from >100 patent offices including the European (EPO), Japanese (JPO), Korean (KIPO), and US patent offices (USPTO) plus WIPO. Each *patent* record provides details on the chemicals referenced in that patent, along with patent title, abstract, application and publication dates, applicant, inventor, and patent classification, but without context about why particular chemicals are mentioned. Individual PubChem *compound* pages contain information about each patent linked to that compound (chemical) in the Patent subsection. A single invention may be described across multiple patent documents (e.g., patent applications, grants, and re-examination certificates) identified with a unique patent identifier suffixed with a code (e.g., A1, A2, B1, B2). The same patent may also be filed in multiple national agencies, which can be grouped together into patent families.

Patent data has been used to prioritize compounds in nontarget environmental studies in a complementary manner to literature counts, aiding in the identification of potential contaminants with known commercial uses.^3^ A recent viewpoint highlighted a concerning upward trend in chemical numbers across databases over time frames much shorter than the typical time for regulatory actions.^4^ The *chemical stripes* visualization included in that viewpoint^4^ sparked extensive debate, drawing significant attention, feedback, and questions from various audiences. The subsequent sonification(5) and accompanying video(6) by J. Perera further intensified the discussion, leaving viewers in a state of shock or deep contemplation. Combining international legislation, patent filing dates, and region information could potentially reveal various trends in patent numbers as well as the effectiveness of regulatory measures and the necessity for timely interventions. However, beyond the general upward trend in chemical and patent numbers, deviating patterns in the stripes visualization were observed for various chemicals, while several potential artifacts and limitations became apparent. This feedback motivated this article, which presents the data, code, and methods behind the *chemical stripes* visualization and corresponding *chemicalStripes* R package^7^ and performs a more detailed analysis of the patent data in PubChem for this particular context, closing with a discussion on the potential and limitations of this data.

## Methods and Materials

### Chemical Stripes Visualization

**Figure 1:**
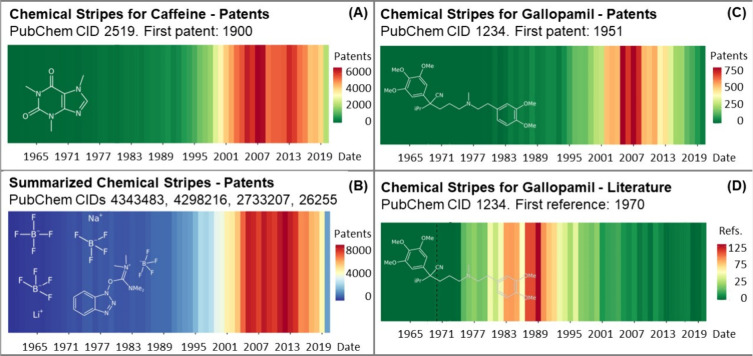
Several examples of Chemical Stripes from 1960 to 2020; structures generated in CDK Depict^14^ are overlaid. (A) Patent stripes for caffeine, showing the typical pattern. (B) Summarized patent stripes for 4 species related to tetrafluoroborate in “colour-blind friendly” mode. (C) Patent stripes. (D) Literature stripes for gallopamil, with atypical patterns.

The open source *chemicalStripes*(7) R package is available on GitLab and was developed to create the *chemical stripes* figure for one or more specific chemicals by PubChem Compound IDs (CIDs). The default is a single CID (Figure 1A), while multiple chemical forms (e.g., salts, stereoisomers) can be handled by inputting multiple CIDs to form summarized stripes (Figure 1B). CIDs can be obtained easily from multiple starting queries using PubChem search functionality.^8^ In addition to the input CID(s), users can specify a date range (default 1960–2023) and mode (patent or literature) and opt for a colorblind friendly version. The patent data is retrieved from the “Depositor Supplied Patent Identifiers” section^9^ of the respective CIDs, while the literature data is retrieved from the “Consolidated References” section.^10^ The default color range is green through yellow to red (Figure 1A,C,D), reproducing a traffic-light scheme distinctive from both the *warming*(11) and *biodiversity stripes*(12) already produced, whereas the colorblind friendly version is blue to red (Figure 1B), very similar to the *warming stripes* (and hence not the preferred default). The chemical stripes function begins by checking package dependencies and loading the necessary libraries. It then retrieves compound information, including the compound name, molecular formula, and a number of patents filed. If patent data is available, this is then downloaded and processed, generating the stripe plot using the ggplot2 package,^13^ both displaying and saving the output as PNG, see examples in Figure 1. For further details see the *chemicalStripes* repository.^7^

### Statistical Analysis

Because a general trend was obvious (Figure 1A,B) with clear outliers (C), the patent data set was analyzed in more detail (the literature data set is much smaller and currently less suitable for these more detailed investigations). Time series clustering was performed to systematically identify outliers, inspired by examples such as the abrupt decrease in patents for gallopamil (Figure 1C). Region-based analysis was performed by inventor region to identify chemicals exclusive to certain regions, or regions with similar patenting activities, while specific trends related to chemical classes such as pharmaceuticals, pesticides, or persistent compounds like per-and polyfluorinated substances (PFAS) were also explored. Additionally, network analysis was employed to uncover relationships and connectivity patterns among chemicals in the patent data set. Specifically, several centrality measures were utilized to assess the importance or influence of chemicals within the constructed network despite their dependency on specific context and sensitivity to small changes in the network structure. Further details are available as open source code in the *ULPatentTrends*(15) repository.

Because the patent data set in PubChem is huge (>4 TB of data), subsets were used to perform these analyses. Distinct topics of interest to the environmental community were chosen to explore further, including legacy pollutants (polychlorinated biphenyls, PCBs), agrochemicals (generally data-rich chemicals), and emerging pollutants of concern (bisphenols and their alternatives, plus PFAS). Curated lists of chemicals (often called “suspect lists”) were extracted from online resources, including the NORMAN Suspect List Exchange (NORMAN-SLE),^16−18^ CompTox,^19−21^ the PubChem PFAS Tree,^22,23^ and the PubChem Compound Table of Contents (TOC) Tree^2^ in the PubChem Classification Browser. The agrochemical lists included the “Agrochemical Information”, “EU Pesticides Data” and “USDA Pesticides Program” sections from the PubChem TOC Tree^2^ and the S28 EUBIOCIDES^24^ list from the NORMAN-SLE. The bisphenol lists included S20 BISPHENOLS^25^ and the alternatives list S97 UBABPAALT^26^ from the NORMAN-SLE, while a polychlorinated biphenyls (PCBs) list was extracted from CompTox.^27^ For PFAS these included the PFAS considered in the PARC project(28,29) (S102 PARCPFAS^30^) and potential persistent, mobile, toxic (PMT) PFAS (S111 PMTPFAS^31^) from the NORMAN-SLE plus four Stockholm Convention lists from the regulatory section of the PubChem PFAS Tree: the initial and updated perfluorooctanoic acid (PFOA) listing,^32^ the initial perfluorohexanesulfonic acid (PFHxS) list^33^ and the proposed C9–C21 long chain perfluoroalkylcarboxylic acids (LC-PFCAs) listing.^34−36^

## Results and Discussion

### Overall and Regional Trends by Chemical Lists

**Figure 2:**
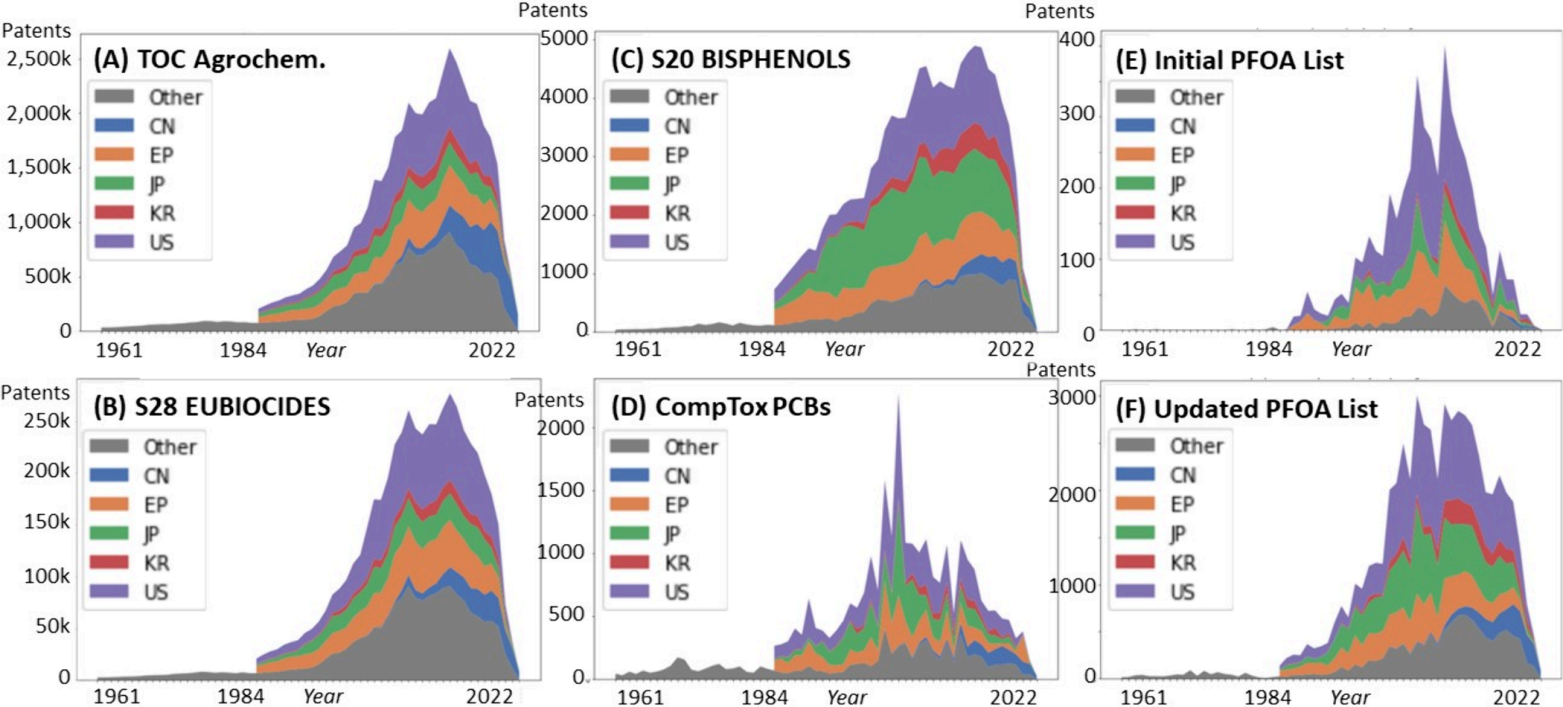
Patent counts for topic-based subsets of chemicals, with regional information. (A) The PubChem Table of Contents (TOC) Agrochemicals category. (B) The NORMAN-SLE S28 EUBIODICES list. (C) The NORMAN-SLE S20 BISPHENOLS list. (D) The CompTox Polychlorinated biphenyls (PCBs) list. (E,F) The initial and updated perfluorooctanoic acid (PFOA) listing in the Stockholm Convention. Purple = US, red = Korea, green = Japan, orange = EU, blue = China, and gray = Other regions. The reasons for the steep decrease in recent years are discussed in the final section of this manuscript.

Information was available for 103 separate regions, with most data generally available for the US, Europe, Japan, China, Korea, and WIPO; the first 5 were chosen for a more detailed regional analysis. One example plot showing the top 20 of the 103 regions is shown in the Supporting Information (SI), Figure S1; additional plots are included in the *ULPatentTrends* repository.^15^ The overall trends in patent numbers over the different lists broken down by the five largest regions (with all others, including world, in the “other” category) reveal varying trends, with six of the 13 examples shown in Figure 2 and explained below. More examples are given in the SI, Figures S2–S6, including some breakdowns per CID, and in *ULPatentTrends*.^15^

The overall trends in the entire agrochemicals category (Figure 2A) and the two EU and US subsets (SI, Figure S2B,C) were quite similar, although the increase in patents from China was less pronounced for the US compared with the EU and overall agrochemical list. A slightly different pattern was observed for EUBIOCIDES (Figure 2B). Figure S3 (SI), a breakdown by compound, shows that this is driven primarily by benzoic acid, propanol, and 2-propanol. Further breakdowns per CID are included in *ULPatentTrends*.^15^ The patterns for S20 BISPHENOLS (Figure 2C) and the alternatives list, S97 UBABPAALT, were almost identical, dominated by bisphenol A (see SI, Figure S4). The plot for CompTox PCBs (Figure 2D) reveals a markedly different pattern with a peak around 2001, potentially due to the impact of the Stockholm Convention signed in 2001 (effective 2004); details by CID are given in SI, Figure S5. This baseline is mainly 2-chlorobiphenyl, with 3,3′,4,4′,5-pentachlorobiphenyl and 3,3′,4,4′-tetrachlorobiphenyl forming the peak around 2000 (see SI, Figure S5). The difference in patent trends for the initial versus updated PFOA listings in the Stockholm Convention (Figure 2E vs F), particularly in the last years (2009–2022), underscore the critical importance of updating regulatory lists, especially in light of the increasing proportion of Chinese patents. Regional plots for all six PFAS lists are included in SI, Figure S6.

### Discovering Important Chemicals in Lists via Centrality Analysis

**Figure 3:**
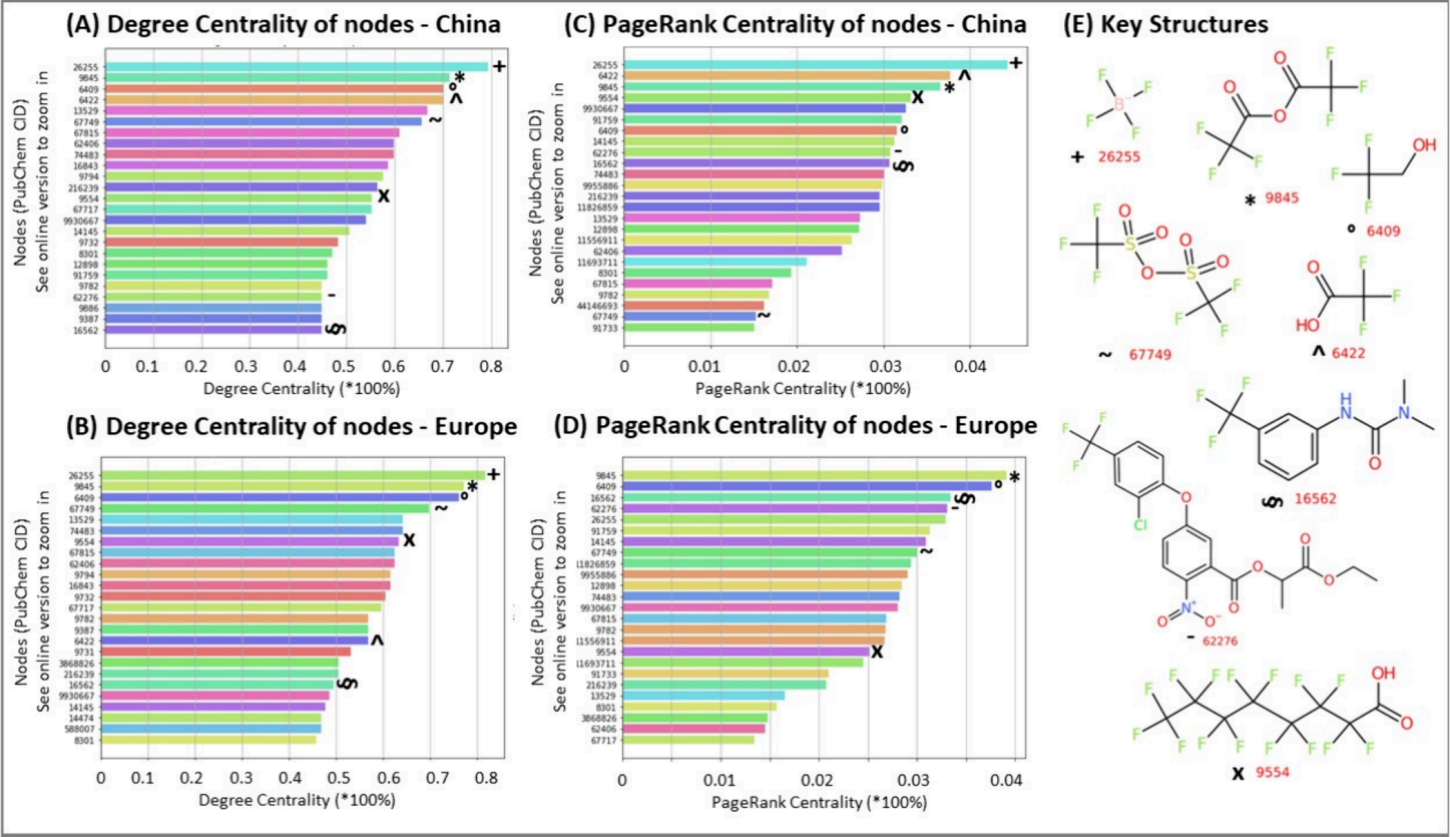
Network analysis on S111 PMTPFAS to find key structures of interest (top 25 nodes). (A) Degree centrality of nodes for China. (B) Degree centrality of nodes for Europe. (C) PageRank centrality of nodes for China. (D) PageRank centrality of nodes for Europe. (E) Key structures, the top four from (A–D), marked on each plot with respective symbols next to the CID.

Networks were constructed for CIDs from chemical lists. Each (weighted) individual network consisted of the CIDs from a specific list as nodes. An edge connecting a pair of CIDs was established if both CIDs were mentioned by one patent. Each edge was further weighted by the number of coappearances of the two CIDs in patents. These individual networks resulted in plots (see SI, Figures S7–S8 and *ULPatentTrends*(15) for examples) that were quite difficult to interpret. More detailed network analysis, shown in Figure 3 (including the US in SI, Figures S9), helped isolate chemicals of particular interest to certain regions.

Figure 3 shows a patent network analysis of the S111 PMTPFAS list, with nodes represented by their degree centrality for China (A) and Europe (B), and their PageRank centrality for China (C) and Europe (D), each featuring the top 25 nodes. This analysis highlights differences in the regional patent activity related to key PFAS compounds. In plots A, B, and C, the top candidate is tetrafluoroborate (CID 26255, see Figure 3E, top left, “+” symbol), which is used for electroplating and as an electrolyte additive for batteries. In the EU PageRank Centrality plot, it ranks only fifth, indicating a relatively lower number of patent applications involving this compound or within this sector compared with other regions or compounds. In plot D, the top compound is trifluoroacetic anhydride (TFAA, CID 9845, “*” in Figure 3E), which is second highest in plots A and B, and third highest in plot C. TFAA serves as a recommended desiccant for trifluoroacetic acid (CID 6422, “∧”), which is fourth in A and second in C, but is less prominent in Europe (B) and absent in D. Trifluoroethanol (CID 6409, “○”), a solvent used in organic chemistry, ranks third in A and B, second in D, and seventh in C. Fluometuron (CID 16562, sideways “§”), a herbicide, is second in D, tenth in C, and lower in both A and B. Lactofen (CID 62276, “–“), another herbicide, appears consistently across A, C, and D but is missing in B. PFOA (CID 9554, “X”), a well-known PFAS compound restricted by the Stockholm Convention since 2019, is present in all four plots, with the highest in Figure 3C (4th place). The differences between the plots underscore how regulatory environments, industrial needs, and research priorities shape the patenting activities related to PFAS compounds in the EU and China (and US; see SI, Figure S9). The presence of compounds like PFOA in both regions highlights ongoing attention to regulated substances, but the specific applications and frequency of patent filings reveal divergent technological focuses and market demands between the two regions. A similar analysis for agrochemicals is included in the SI, Figure S10. Both examples show how investigating the patent data on various lists of chemicals could help isolate “stand-out” trends in chemical activity and act as (or help support) a potential early warning system for up and coming action.

The regional analysis of the patent data set (see SI, Figures S9 and S10) included CIDs that are unique to specific regions, such as agrochemicals present only in China, Europe, or the US, or those unique to only two regions, such as China and Europe but not US. The analysis covering all possible regional subsets and can be found in *ULPatentTrends*;^15^ custom queries can be formed from the CID lists. Examples of the agrochemical list are given in the TOC graphic.

### Potential and Limitations of Patent Data

There are several challenges and limitations associated with analyzing the chemical stripes visualizations and patent data set. The patent data set, while comprehensive, may not always be current or complete and seems to contain historical depositions that have been discontinued (potentially partially explaining some “blips” seen around 2007 and 2016), posing significant hurdles for accurate data (and trend) analysis. Extracting chemical information via image recognition or text mining from patents is challenging because older patent documents are lower quality and are thus quite noisy and error prone.^37^ The data set in PubChem is reliant on the information provided by depositors and is mapped to CIDs during deposition based on the data received. Often, chemicals mentioned in the introduction of patents are not actually used in the application, leading to potential misinterpretation of their relevance, while any chemicals that happen to appear in Markush structures defined for drug discovery purposes may be overrepresented. Chemicals can be mistakenly identified due to their appearance in unrelated contexts, such as being part of an inventor’s name. Such an error, reported for the 1913 patent linked to PFOS (US-1257524-A), based on a figure in the earlier viewpoint, led to a suggestion to use a higher threshold in the cutoff applied to the scoring of name recognition in future applications. The two earliest PFOS patents currently in PubChem (as of 29 June 2024) are due to misrecognized names: US-1257524-A was invented by Adolf **Pfos**er, while US-2290705-A was invented by Wilhelm **Pfos**t, the first genuine PFOS patent is 1953 (see SI, Figure S11). Additionally, recent patents for PFOS may, for example, reflect new treatment technology, not PFOS production or use. The tabular view of the patent data in PubChem allows a quick check of the linked patents, assisting with rapid verification (SI, Figure S12); this can also be downloaded for offline use.

The timeliness of patent data is a major issue. Patent records from recent years, particularly post-2020, are often incomplete, which can skew analysis and trends; it can take 1–2 years for the data to filter through more completely. This is exacerbated by the use of the “priority date”, which can add an 18 month delay from filing but is the preferred date for the majority of PubChem users. This gap highlights the need for rapid updates of data sources to ensure that recent innovations and filings are accurately represented. The accuracy of the data set is heavily reliant on the efficiency and precision of literature mining and image recognition algorithms, which is an area of active research.^37−40^ Adjustments to the extraction workflows can result in the exclusion of several patents from the data set or the omission of information such as priority dates (which, if absent, cannot be included in the chemical stripes).

Future research directions could include exploring different patent sources, such as using AI applications such as DeepSearch by IBM to collect and curate documents, which may offer more reliable and comprehensive data. Refining the data mining tools would also help in managing and interpreting the patent data set more effectively.

The chemical stripes package (*ChemicalStripes*^7^) and Jupyter notebooks (*ULPatentTrends*^15^) provided as part of this work are designed to help environmental researchers explore the possibilities of using patent data to address their environmental questions. This will help facilitate a deeper understanding of regional trends, regulatory impacts, and innovation landscapes in the chemical sector as well as limitations in this data. Users are encouraged to report any artifacts because such feedback is crucial for refining the data set and approaches.^41^ Incorporating more sophisticated classification methods in data extraction may help further enhance the accuracy and usability of these data in the future.

## Data Availability

Dagny Aurich, Emma Schymanski, Flavio De Jesus Matias, Paul Thiessen, Jun Pang. Revealing Chemical Trends: Insights from Data-Driven Visualization and Patent Analysis in Exposomics Research. 2024. ChemRxiv. 10.26434/chemrxiv-2024-6jkxv (accessed 2024-08-22). Code is available online at https://gitlab.com/uniluxembourg/lcsb/eci/chemicalstripes (Chemical Stripes) and https://gitlab.com/uniluxembourg/lcsb/eci/ULPatentTrends (ULPatentTrends notebooks).

## Supporting Information Available

Brief text describing the repository, figures showing patent count by region, regional patent counts for agrochemicals, biocides, bisphenols, PCBs, PFAS, connected and disconnected networks, centrality results for China, EU, and US for PFAS and Agrochemicals, screenshots of verifying patent information in PubChem (PDF)
