# Supplementary material for: Revealing Chemical Trends: Insights from Data-Driven Visualization and Patent Analysis in Exposomics Research
Source: Environ Sci Technol Lett. 2024 Aug 30;11(10):1046–52. doi: 10.1021/acs.estlett.4c00560 (PMC11465647; doi:10.1021/acs.estlett.4c00560)
Supplement: Supplementary file 1 — ez4c00560_si_001.pdf [file ez4c00560_si_001.pdf]

Supporting Information for Revealing Chemical Trends: Insights from Data-Driven Visualisation and Patent Analysis in Exposomics Research

Dagny Aurich*[1], Emma L. Schymanski*[1], Flavio de Jesus Matias[1,2], Paul A. Thiessen[3], Jun Pang[2]

[1] *Luxembourg Centre for Systems Biomedicine (LCSB), University of Luxembourg, 6 Avenue du Swing, Belvaux L-4367, Luxembourg.*

[2] *Faculty of Science, Technology and Medicine (FSTM), University of Luxembourg, 6 Avenue de la Fonte, L-4364 Esch-sur-Alzette, Luxembourg.*

[3]*National Center for Biotechnology Information, National Library of Medicine, National Institutes of Health, Bethesda, MD 20894, USA.*

ORCIDs: DA: 0000-0001-8823-0596; ELS: 0000-0001-6868-8145; FdJM: 0009-0008-8585-7690; *PAT: 0000-0002-1992-2086; JP: 0000-0002-4521-4112.*

*Corresponding & co-first authors: dagny.aurich@uni.lu, emma.schymanski@uni.lu

Contents

Supplementary Text

Brief supplementary text is given on Page 2.

Supplementary Figures

Figure S1	Patent counts by region of origin	2
Figure S2	Patent counts for topic-based subsets: Agrochemicals	3
Figure S3	Patent counts for S28 EUBIOCIDES – per compound breakdown	3
Figure S4	Patent counts for the bisphenol lists – with compound breakdown	4
Figure S5	Patent counts for the PCBs – with compound breakdown	4
Figure S6	Patent counts for topic-based subsets: PFAS	5
Figure S7	Connected network examples	5
Figure S8	Disconnected network example – S102 PARCPFAS	6
Figure S9	Network analysis on S111 PMTPFAS – China, Europe and US	6
Figure S10	Network analysis on TOC Agrochemicals – China, Europe and US	7
Figure S11	Screenshots of patent information for PFOS - artefacts	8
Figure S12	Verifying linked chemical information in patent documents	8

Supplementary Text

The code to create the figures in the main text and this supplementary information is available on GitLab, with a README file describing the structure of the repository with cross reference to several of the figures:

https://gitlab.com/uniluxembourg/lcsb/eci/ULPatentTrends/-/tree/main#repository-contents

Supplementary Figures

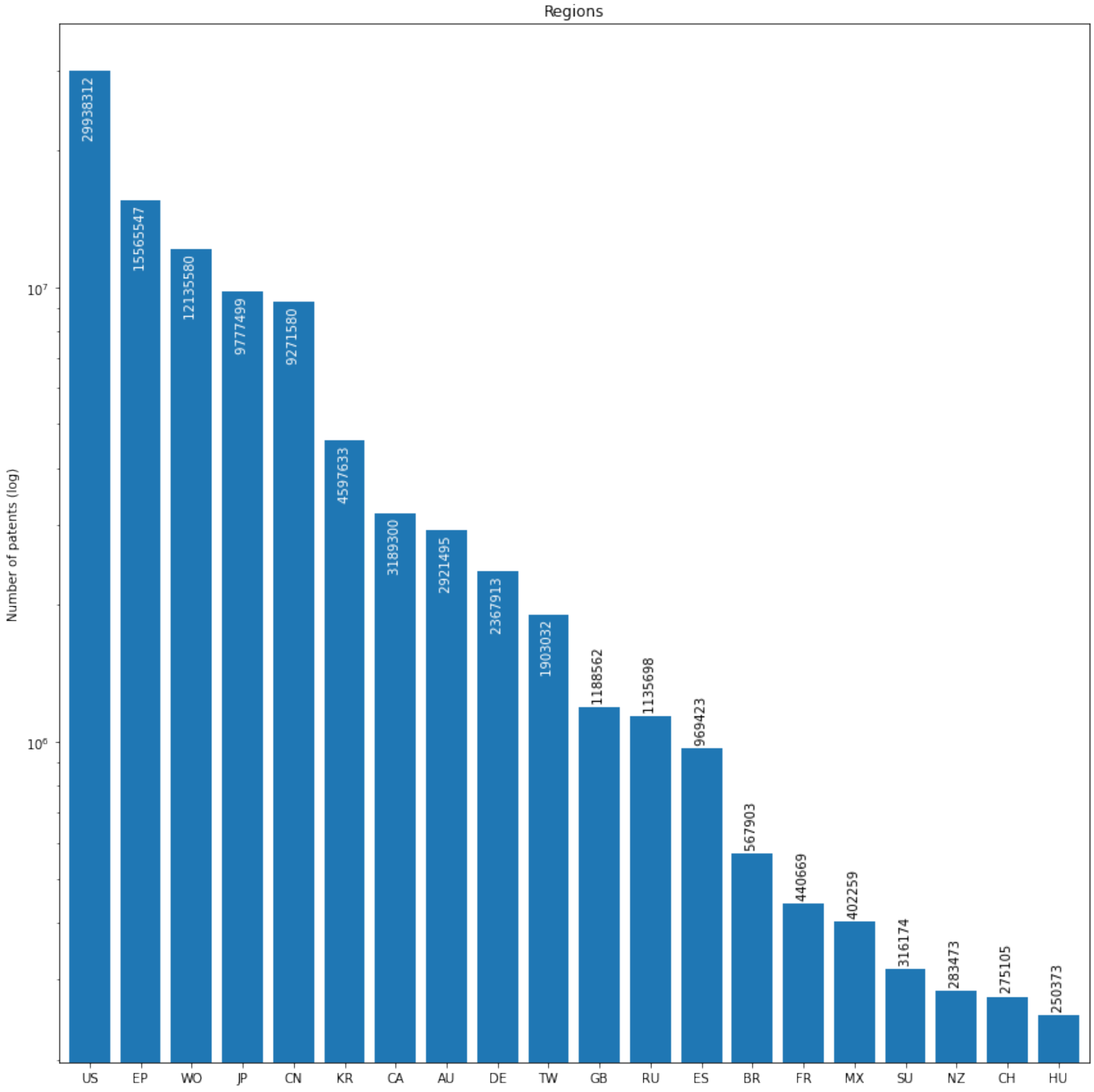

Figure S1: Patent counts by region of origin for 100M rows of the patent dataset. The top 20 of 103 regions are shown. Image extracted from visualize-CID-Patent.ipynb. Note that this distribution changes for patent families and for other row counts, this equivalent plot by family is included in visualize-ID-Date-Family.ipynb; other plots in result-100.000.000-rows.ipynb and result-250.000.000-rows.ipynb.

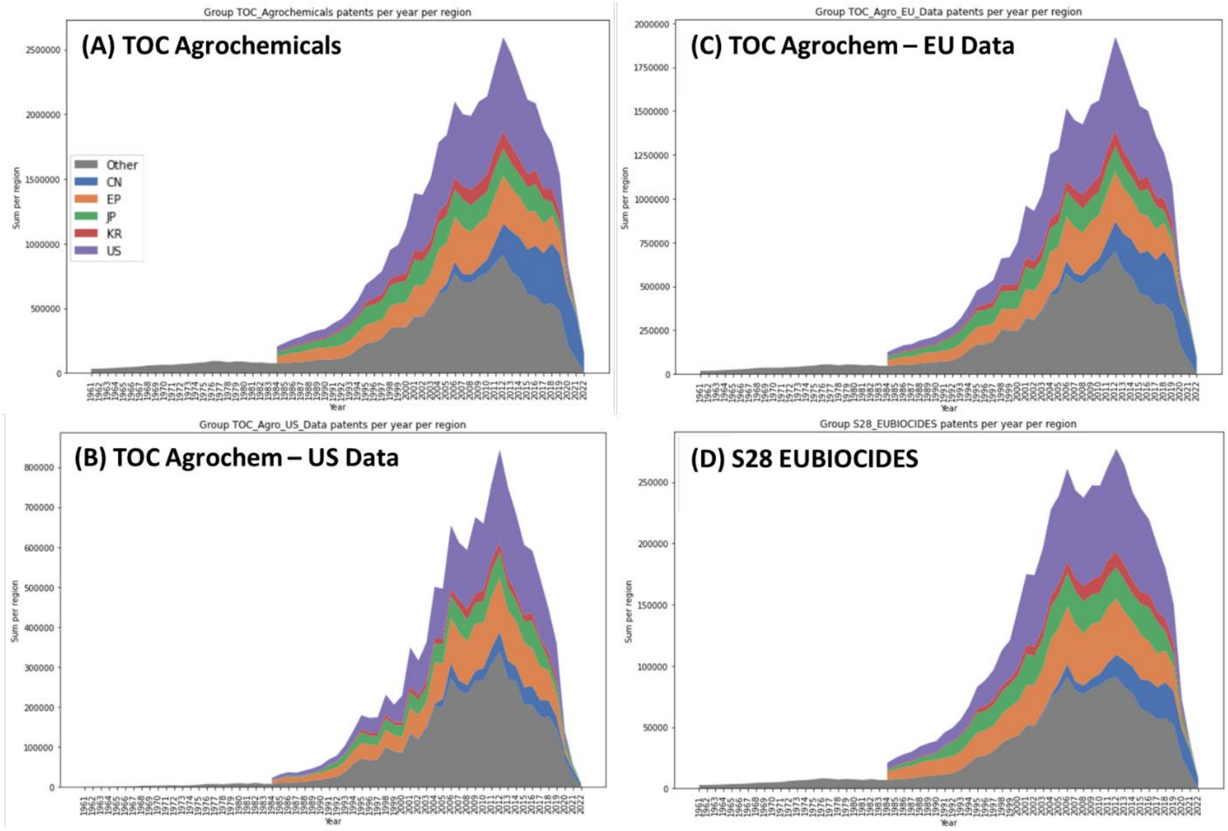

Figure S2: Patent counts for topic-based subsets of chemicals, with regional information - Agrochemical subset. (A) The PubChem Table of Contents (TOC) Agrochemicals category. (B) The TOC Agrochemicals category subset "USDA Pesticides Program". (C) The TOC Agrochemicals category subset "EU Pesticides Data". (D) The NORMAN-SLE S28 EUBIOCIDES list.

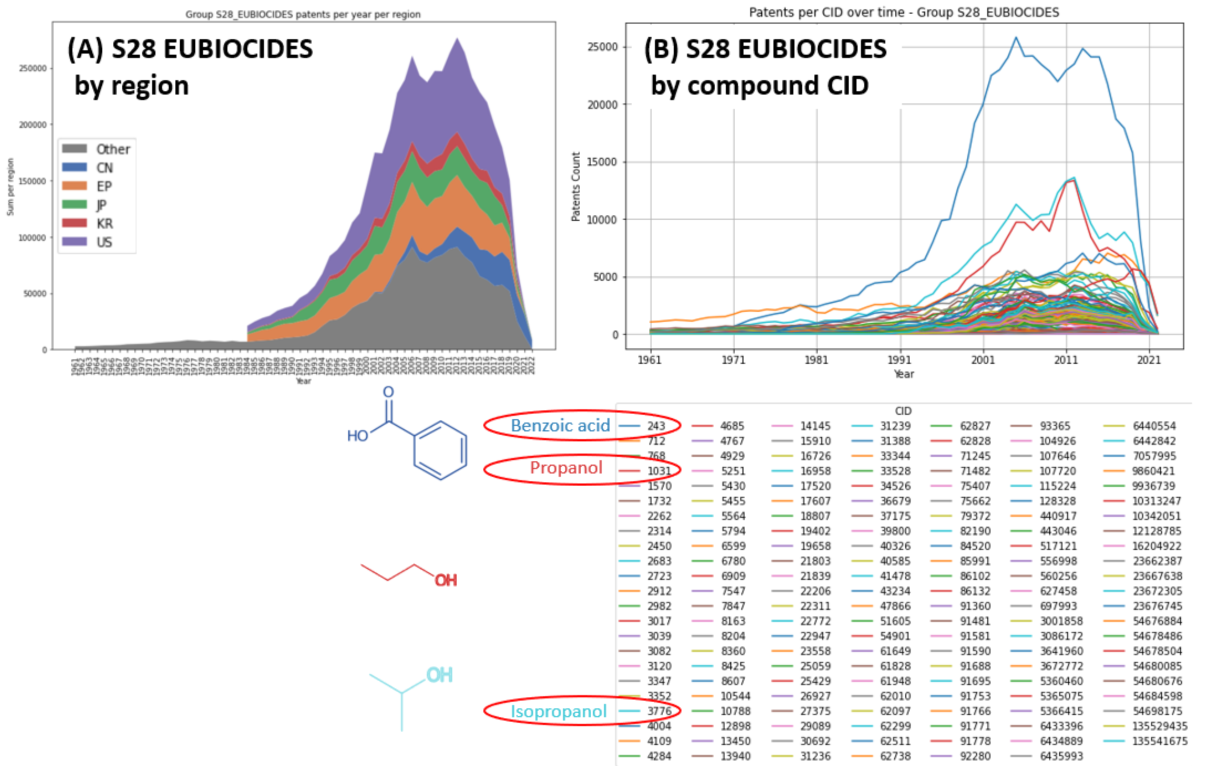

Figure S3: Patent counts for the S28 EUBIOCIDES list per compound (represented by the PubChem Compound Identifier CID). Top: Left: Image from Figure S1(D). Top Right: breakdown by CIDs, listed bottom right. Bottom left: structures for top 3 CIDs by patent count: benzoic acid (CID 243), propanol (CID 1031) and isopropanol (CID 3776).

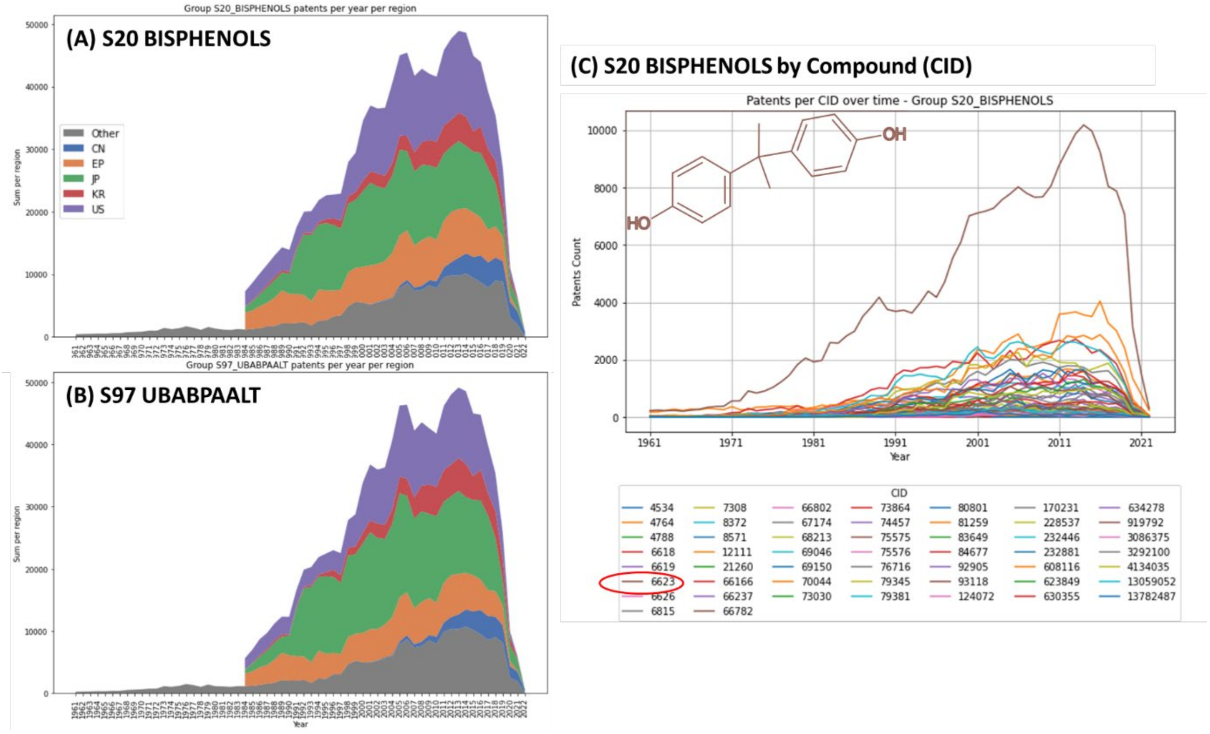

Figure S4: Patent counts for the bisphenol lists. (A) S20 BISPHENOLS by region. (B) S97 UBAPBAALT (BPA Alternatives list from UBA) by region. (C) The S20 BISPHENOLS list per compound (represented by the PubChem Compound Identifier CID), dominated by bisphenol A (BPA), CID 6623 (circled). Inset: structure of BPA.

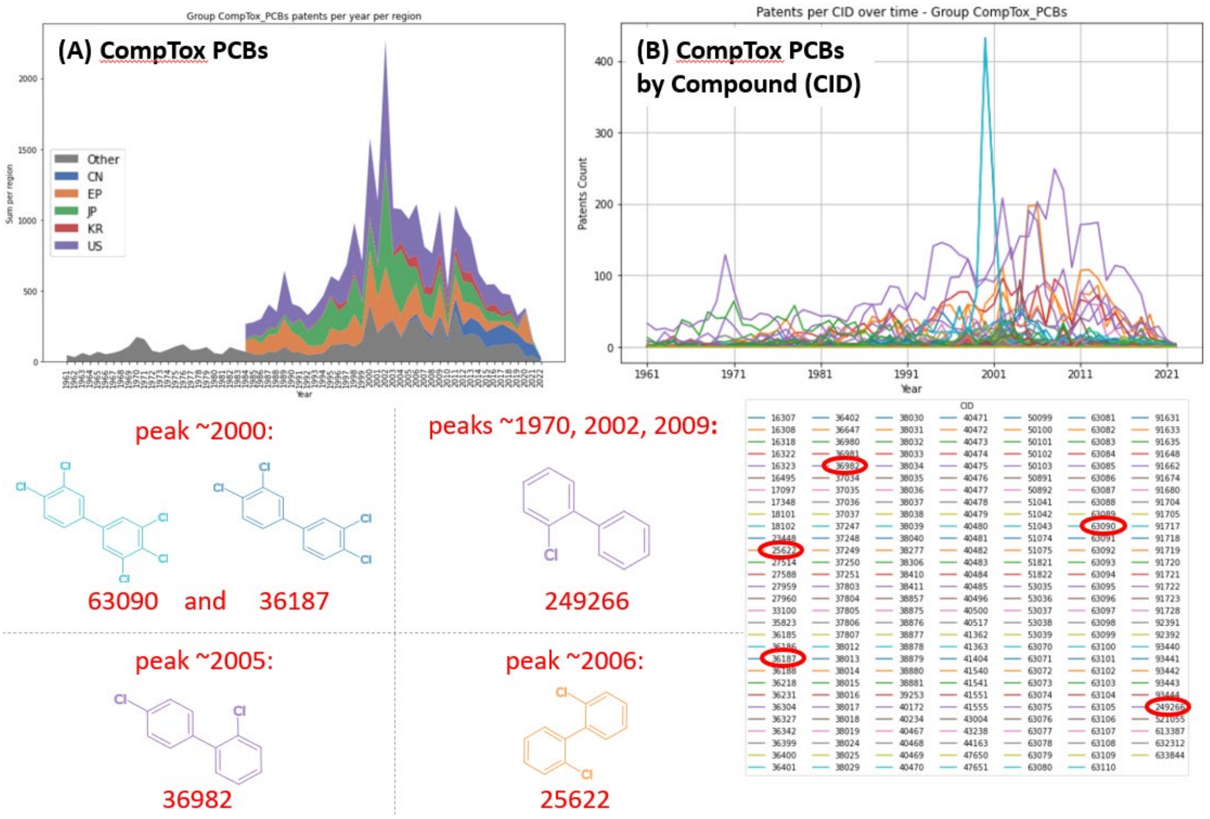

Figure S5: Patent counts for the PCBs. (A) CompTox PCBs by region. (B) CompTox PCBs by Compound (CID). Inset: structures of stand-out PCBs.

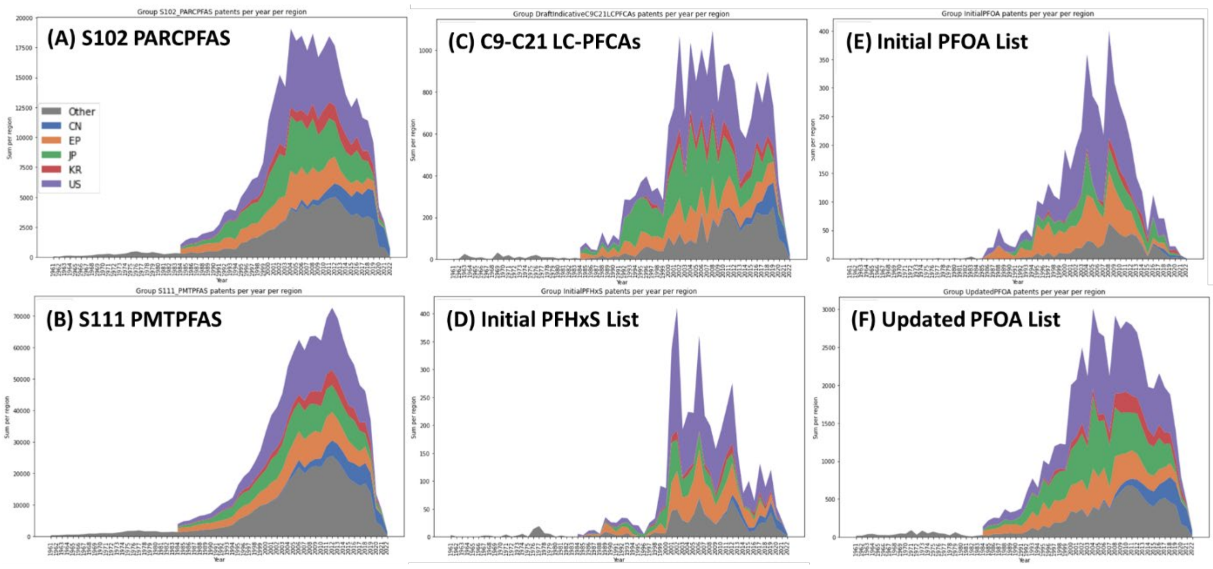

Figure S6: Patent counts for topic-based subsets of chemicals, with regional information - PFAS subset. (A) The NORMAN-SLE S102 PARCPFAS list. (B) The NORMAN-SLE S111 PMTPFAS list. (C) The C9-C21 LC-PFCAs (D) initial PFHxS (E) Initial PFOA and (F) Updated PFOA listing from the Stockholm Convention regulatory collection on the PubChem PFAS Tree. For further details see main text.

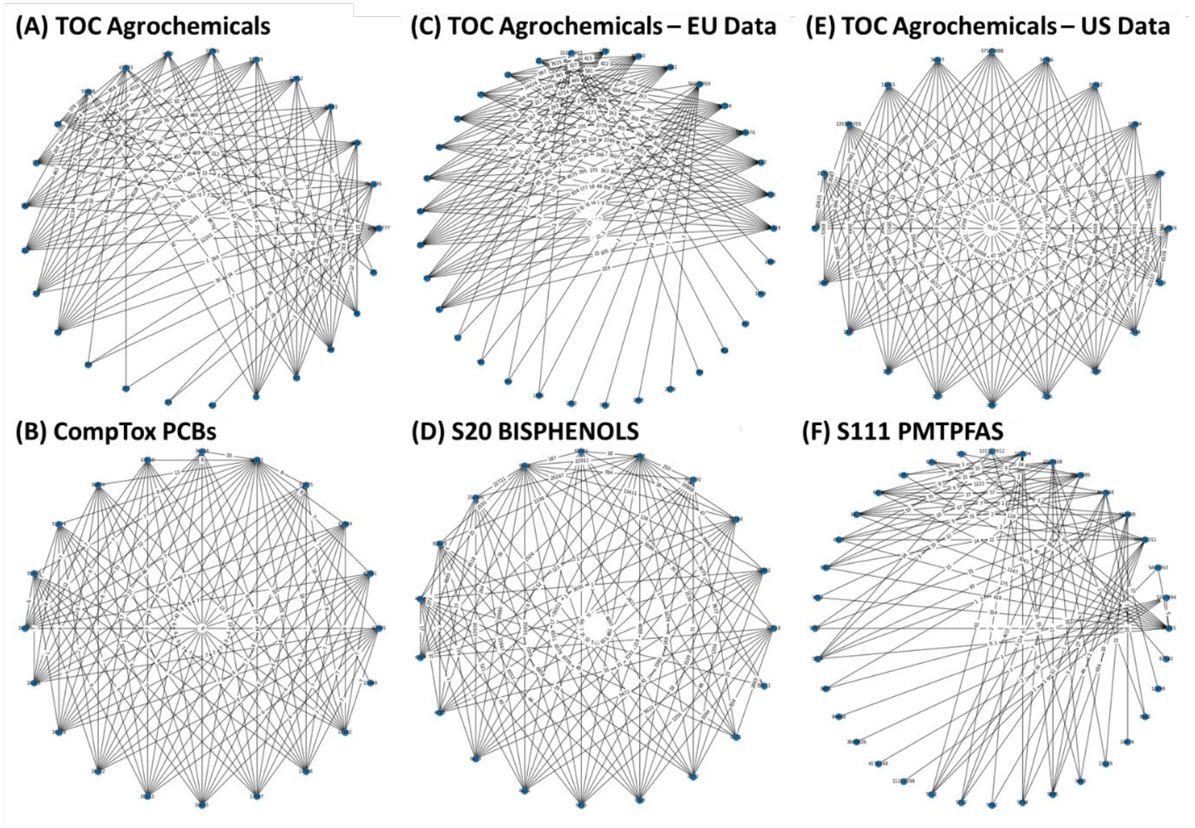

Figure S7: Connected network examples for several lists using 10 CIDs with 10 edges per CID, taken in top order. (A) The TOC Agrochemicals list; (B) CompTox PCBs, (C) TOC Agrochemicals – EU data; (D) S20 BISPHENOLS; (E) TOC Agrochemicals – US data and (F) S111 PMTPFAS. Further examples of connected networks are given in the GitHub repository.

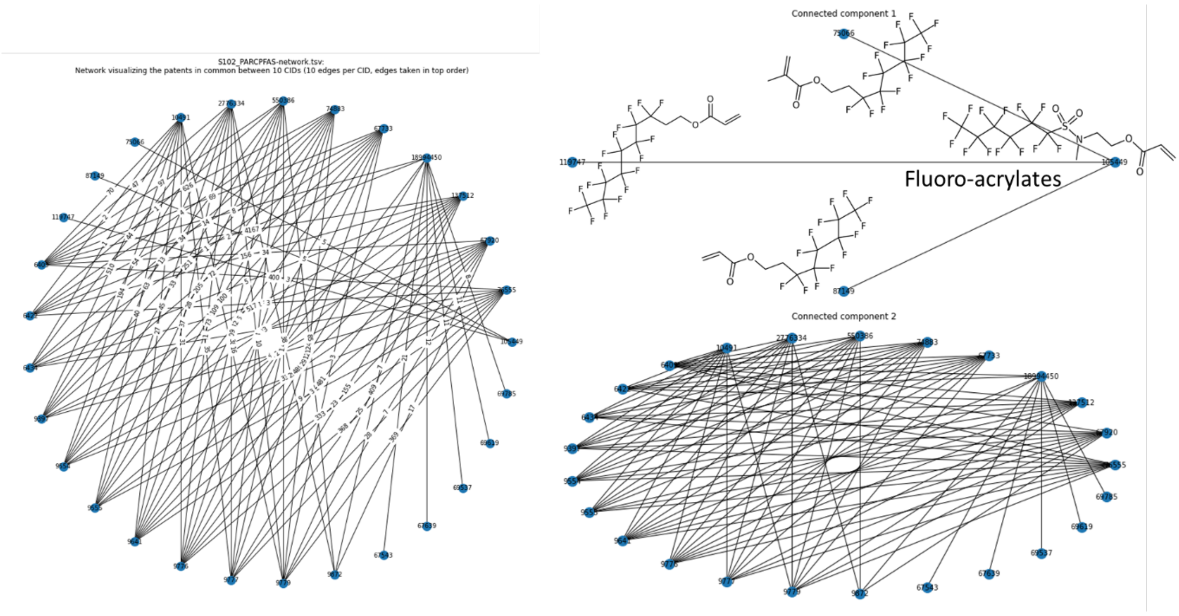

Figure S8: Example of a disconnected network for S102 PARCPFAS using 10 CIDs with 10 edges per CID, taken in top order.

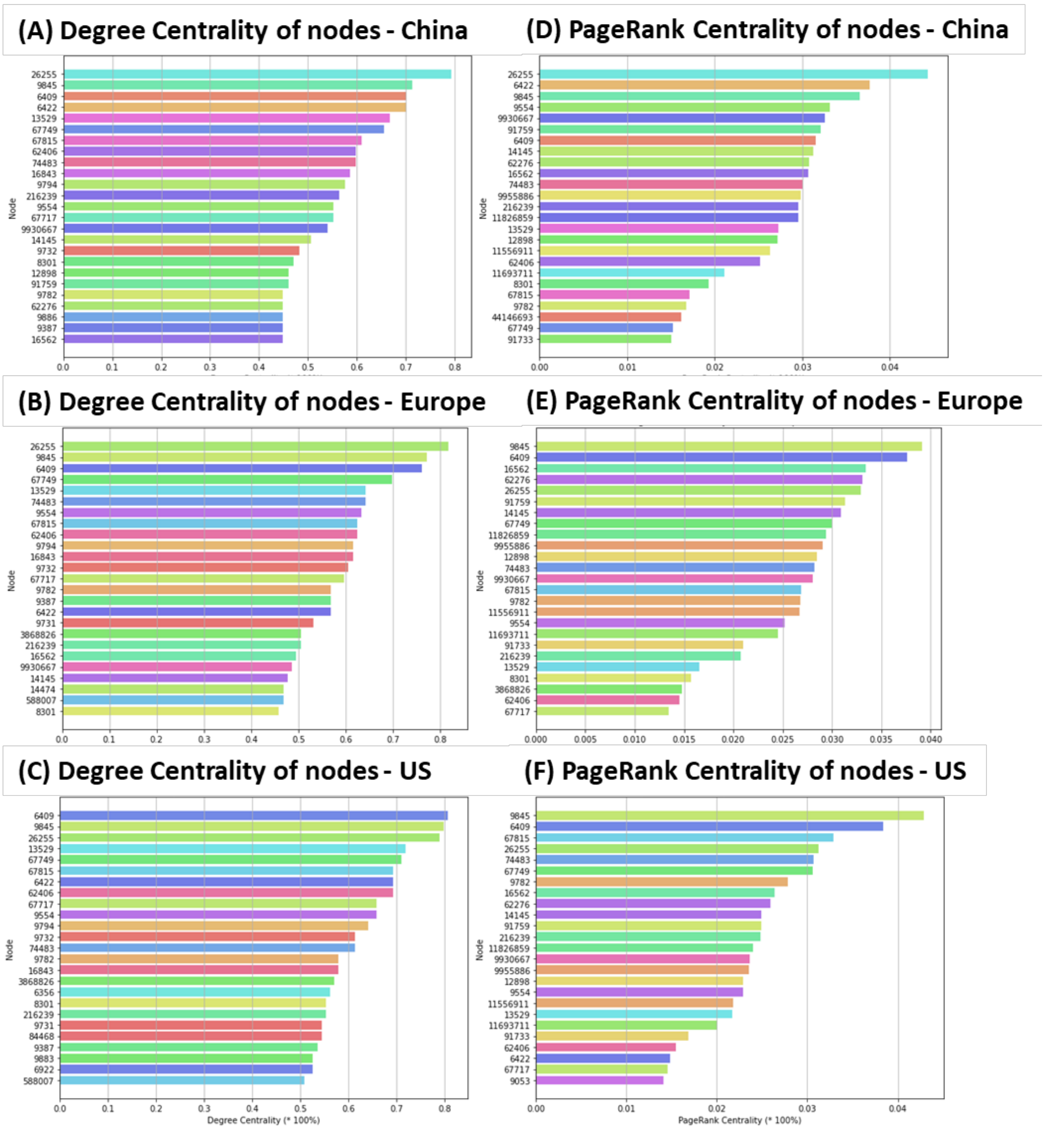

Figure S9: Network analysis on S111 PMTPFAS. (A-C) Degree centrality of nodes for China, Europe and the US, respectively. (D-F) PageRank centrality of nodes for China, Europe and the US, respectively. Further details in the main text.

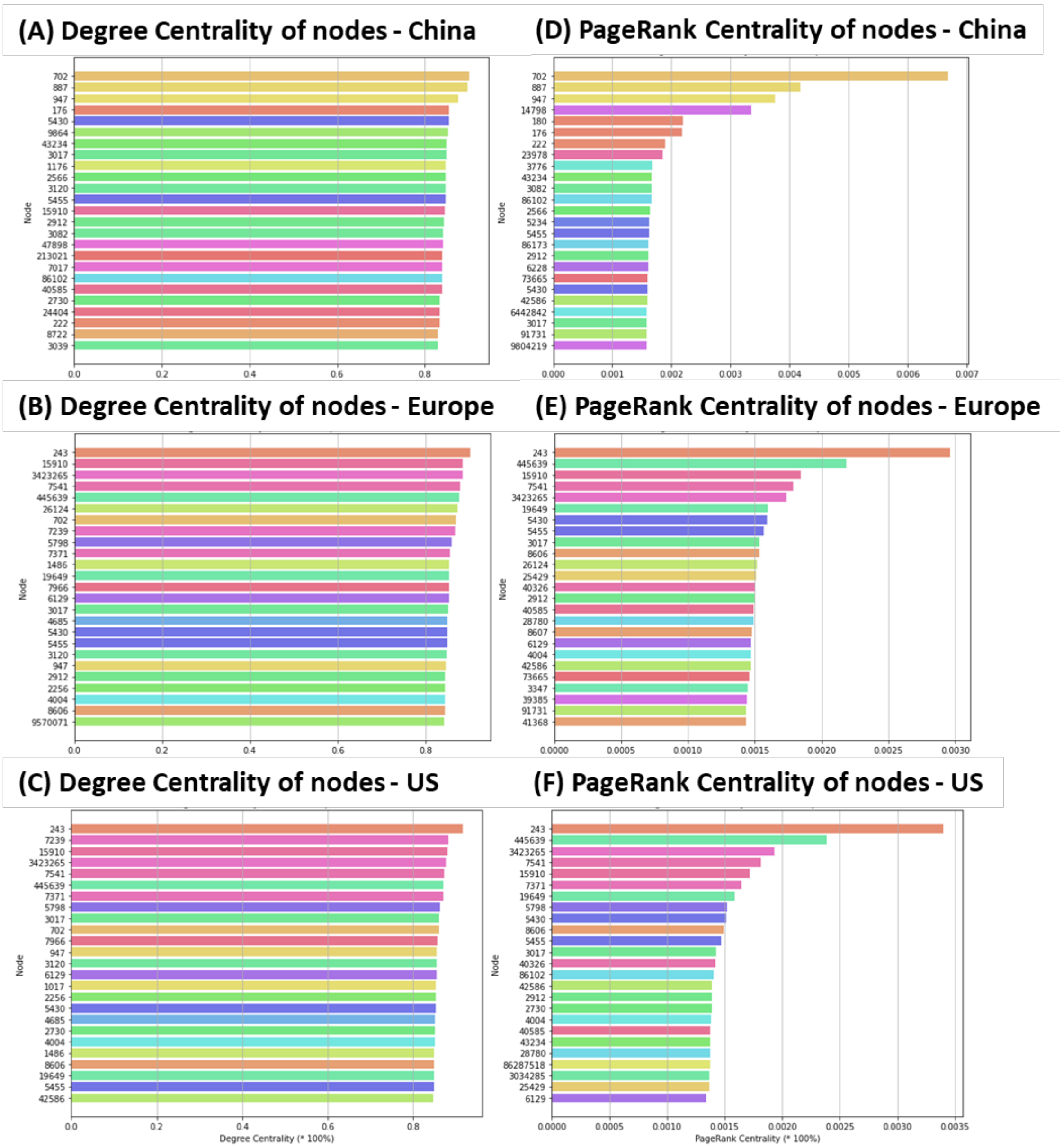

Figure S10: Network analysis on TOC Agrochemicals. (A-C) Degree centrality of nodes for China, Europe and the US, respectively. (D-F) PageRank centrality of nodes for China, Europe and the US, respectively. See text for details.

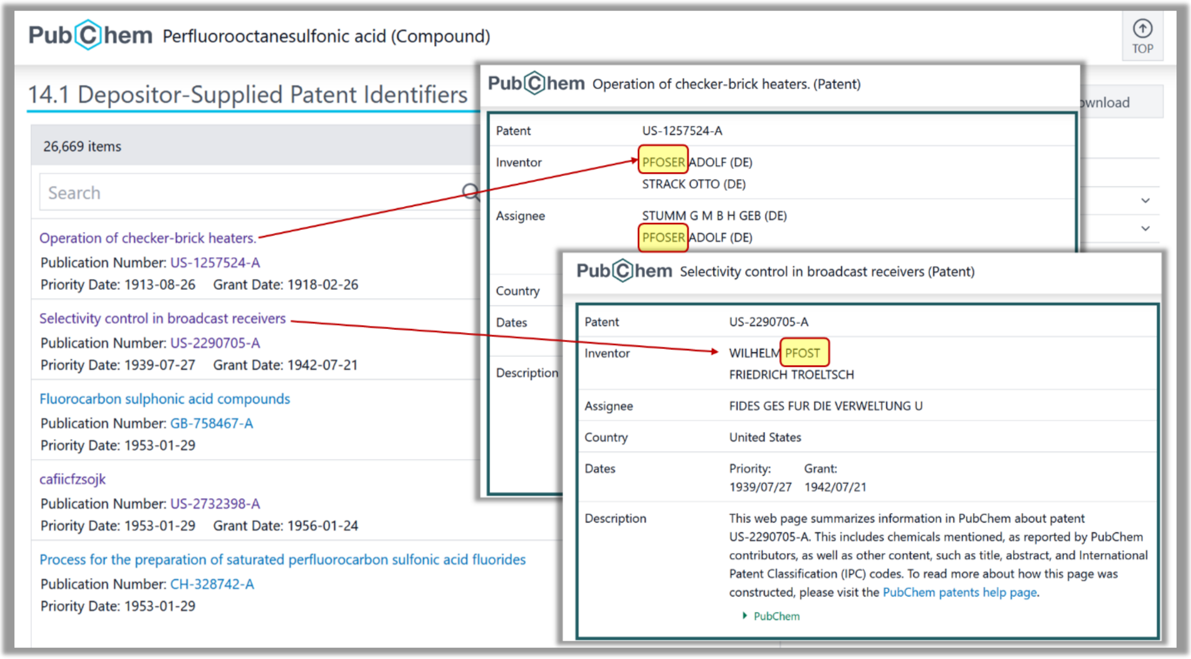

Figure S11: Screenshots of the patent information for PFOS obtained through the PubChem interface on 29 June 2024 using the URL https://pubchem.ncbi.nlm.nih.gov/compound/74483#section=Depositor-Supplied-Patent-Identifiers (sort by "priority date oldest") and the corresponding patent information for patents US-1257524-A and US-2290705-A.

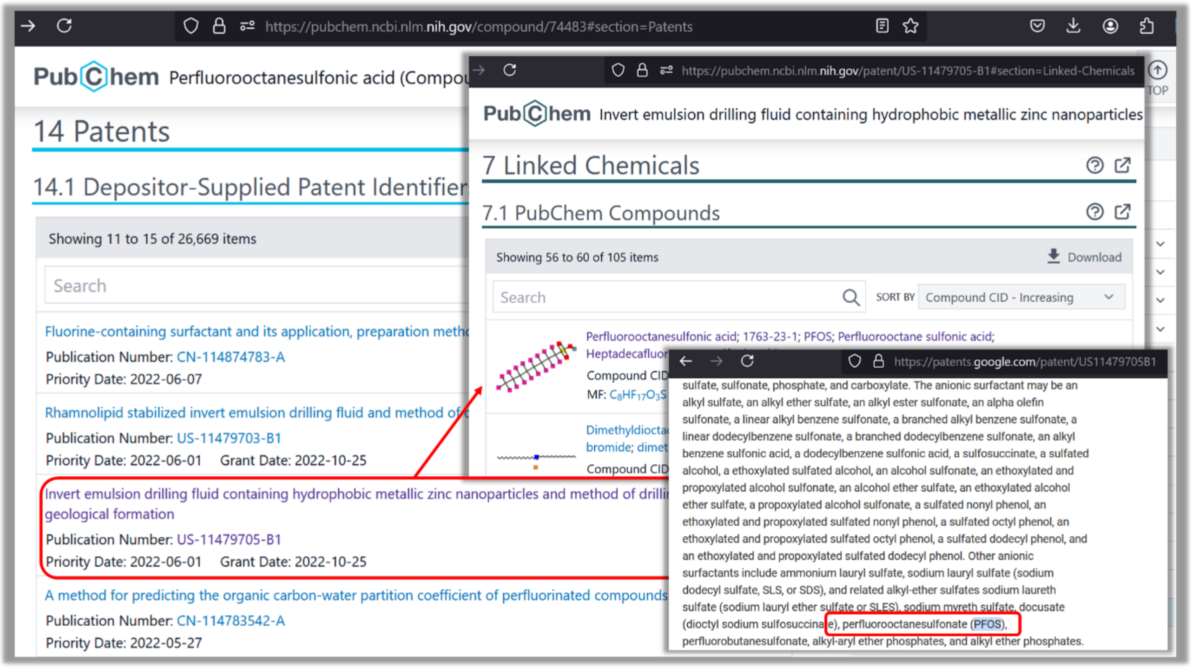

Figure S12: Verifying linked chemical information in patent documents. Background: patent table for PFOS compound entry in PubChem (https://pubchem.ncbi.nlm.nih.gov/compound/74483#section=Patents). First inset: corresponding patent page for patent US-11479705-B1 (https://pubchem.ncbi.nlm.nih.gov/patent/US-11479705-B1#section=Linked-Chemicals). Second inset: the corresponding Google Patent page where PFOS is mentioned in the text (https://patents.google.com/patent/US11479705B1).
